# The feasibility and utility of grocery receipt analyses for dietary assessment

**DOI:** 10.1186/1475-2891-5-10

**Published:** 2006-03-30

**Authors:** Sarah Levin Martin, Teresa Howell, Yan Duan, Michele Walters

**Affiliations:** 1 Morehead State University, College of Education, Department of Health, Physical Education, and Sport Sciences at the time of this study, USA; 2 Associate Professor of Nursing, Morehead State University, 150 University Blvd. Box 715, Morehead, Kentucky 40351, USA

## Abstract

**Objective:**

To establish the feasibility and utility of a simple data collection methodology for dietary assessment.

**Design:**

Using a cross-sectional design, trained data collectors approached adults (~20 – 40 years of age) at local grocery stores and asked whether they would volunteer their grocery receipts and answer a few questions for a small stipend ($1).

**Methods:**

The grocery data were divided into 3 categories: "fats, oils, and sweets," "processed foods," and "low-fat/low-calorie substitutions" as a percentage of the total food purchase price. The questions assessed the shopper's general eating habits (eg, fast-food consumption) and a few demographic characteristics and health aspects (eg, perception of body size).

Statistical Analyses Performed. Descriptive and analytic analyses using non-parametric tests were conducted in SAS.

**Results:**

Forty-eight receipts and questionnaires were collected. Nearly every respondent reported eating fast food at least once per month; 27% ate out once or twice a day. Frequency of fast-food consumption was positively related to perceived body size of the respondent (p = 0.02). Overall, 30% of the food purchase price was for fats, oils, sweets, 10% was for processed foods, and almost 6% was for low-fat/low-calorie substitutions. Households where no one was perceived to be overweight spent a smaller proportion of their food budget on fats, oils, and sweets than did households where at least one person was perceived to be overweight (p = 0.10); household where the spouse was not perceived to be overweight spent less on fats, oils, and sweets (p = 0.02) and more on low-fat/low-calorie substitutions (p = 0.09) than did households where the spouse was perceived to be overweight; and, respondents who perceived themselves to be overweight spent more on processed foods than did respondents who did not perceive themselves to be overweight (p = 0.06).

**Conclusion:**

This simple dietary assessment method, although global in nature, may be a useful indicator of dietary practices as evidenced by its association with perceived weight status.

## Introduction

The obesity epidemic is burgeoning in this country; caloric balance nationwide is tipped toward weight gain. Survey evidence suggests that physical activity levels have remained fairly constant, [1] although there is some evidence that the number of persons reporting no leisure-time physical activity has declined.[2] It was recently reported that "sweets and desserts," soft drinks, and alcohol comprise almost 25% of all calories consumed by Americans.[3] Caloric balance (energy intake = energy expenditure) for weight maintenance is indisputable, but the measurement of both energy intake and energy expenditure is flawed. At the population level, the most common and feasible way to collect behavioral data is through self-report. The literature on the limitations of self-report of diet is extensive. [4-7] Certain food items are more likely to be under-reported than others and dietary intake is highly variable day to day;[8] addressing these issues requires that multiple measures be made over time, which can be burdensome to the participants.

Given the global nature of the obesity epidemic, and the inherent problems with dietary assessment of dietary intake, one might argue that broader measures or proxies for dietary intake are needed and would provide useful information. For example, researchers have discovered that eating patterns, such as whether a person eats breakfast, may be related to body mass index (Pereira MA, Van Horn L, Slattery M, Jacobs DR Jr, Ludwig DS, Reported Breakfast Habits and Incidence of Obesity and the Insulin Resistance Syndrome in Young Black and White Adults: The CARDIA Study American Heart Association's annual conference, March 6, 2003 Miami FL [Unpublished data]) while more exact measures (e.g., 24-hour recall) could lead to spurious results as concluded by Summerball and colleagues.[9] Another recent study has shown that the local food environment (e.g., presence of supermarkets) is associated with dietary intake.[10] Fast-food consumption has also been implicated in the obesity epidemic.[11,12]

We are not aware of any studies in the U.S. that have investigated the utility of a grocery receipt as a proxy for dietary assessment; though most would agree that shopping habits largely reflect eating habits. Cheadle and colleagues[13,14] showed that the grocery store environment can affect individual diets and have argued for using environmental indicators, such as grocery surveys, as evaluation tools.[15] We conducted the present study to determine whether a simple data collection methodology including analysis of grocery receipts and a question about frequency of fast-food consumption could be used as a global measure of dietary assessment by examining its concurrent validity as determined by its possible association with perceived body size.

## Methods

### Subjects

A total of 50 shoppers were recruited from grocery stores in eastern Kentucky to participate (49 women and 1 man). Based on observation, all respondents were between the ages of 20 and 40 years; and as representative of the region, all respondents were white, non-Hispanics. The region's population is characterized by low income levels: according to data from the Bureau of Economic Analysis, the per capita income of the surrounding county in 2002 was $19,309. That for the entire state of Kentucky was $25,494, which is still lower than the per capita income of the nation as a whole ($30,906).[16] More than 20% of the county's population live below the poverty level, compared with about 12% nationwide.[17]

### Procedures

In a cross-sectional design, trained data collectors approached adults (~20 to 40 years of age) at local grocery stores and asked whether they would volunteer their grocery receipts and answer a few questions for a small stipend ($1). Only shoppers with a full cart were approached to increase the likelihood of sampling representative items; the shoppers were asked whether the shopping list was representative of what they often ate. If not, the shopper was excluded from the final analyses (n = 2). The shoppers' decision to participate was entirely voluntary. The data collector explained the purpose of the study and informed consent was inferred by the shopper's participation. Names of participants were not recorded. The data were collected during the fall semester of 2002 by graduate students in an introductory epidemiology class. Institutional review board approval was obtained from the local university (October 2002).

### Instrument and variables

The questionnaire was a single page, front and back (See Appendix). The questions assessed the shopper's general eating habits (eg, fast-food consumption) and a few demographic characteristics and health aspects (eg, health status and perceived body size as indicated on silhouette drawings). Respondents answered for themselves and their family members on the drawings. Perceived overweight status was assigned to the silhouette drawing number five and higher. Previous studies have shown that silhouettes are related to measured body mass index (BMI) and can be used to classify the weight status of adults[18,19] and adolescents.[20] For frequency of fast-food consumption, respondents could answer times per day, week, or month. The fast-food responses were transformed into frequency per month by taking the product of times per day × 30.3 and that of times per week × 4.35.

Grocery receipts were coded by the 4 authors. The grocery data were divided into 3 categories: "fats, oils, and sweets" as classified on the US Department of Agriculture Food Guide Pyramid,[21] including sugared soft drinks, candy, regular potato chips, cookies, mayonnaise, oil, butter, and lard; "processed foods," that is, food prepared with hydrogenated fats or cured meat such as bacon, hot dogs, bologna, and regular frozen meals; and "low-fat/low-calorie substitutions," such as skim milk, diet soda, and baked potato chips. Percent of the total food purchase was calculated for each of the 3 coded categories; nonfood items, cigarettes, and alcohol were excluded from the total purchase.

### Statistical analyses

Descriptive and analytic analyses were conducted in SAS version 8 (SAS Institutute Inc, Cary). Means and frequencies were calculated to describe the population. Given the skewed distribution of much of the data and the small sample size, nonparametric tests were used. It was hypothesized that purchase of fats, oils and sweets and processed food would be positively associated with perceived overweight, and that low-fat/low-calorie substitutions would be negatively associated with perceived overweight; hence the Wilcoxon two-sample, one-sided, exact test was used to determine if there was a relationship between allocation of food purchases for each of the 3 categories (fats, oils, and sweets %, processed foods %, and low/fat/low-calories substitutions %) and perceived body size of: (1) the respondent (overweight versus not overweight), (2) the respondent's spouse, and (3) anyone in the household (no one overweight versus anyone overweight). The same 3 comparisons were run to determine whether there was a relation between frequency of fast-food consumption and perceived body size. The number of respondents varied per model depending according to the family structure. Given the small sample size an alpha of 0.10 was selected to determine statistical significance.

## Results

**Figure 1:**
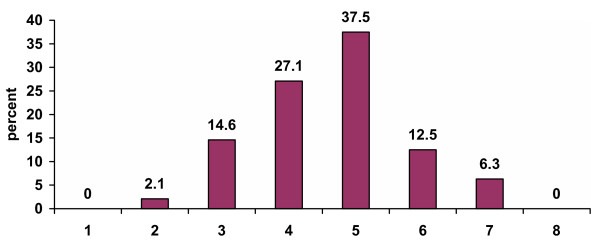
Respondents' perceived body size in accordance with silhouette drawings Legend. A 5 or higher on the scale of 1–8 was defined as perceived overweight (see Appendix).

Forty-eight grocery receipts were collected; 98% (47 of 48) of the respondents were women. Characteristics of the shopping trip, health status, and health-related behaviors of the sample are summarized in Table 1; of note, nearly all respondents (44 of 48; 91.7%) were shopping for themselves and others in the household. The respondents' perception of their body size is summarized in Figure 1; 56% (27 of 48) of the respondents selected a silhouette drawing that was overweight. Respondents also reported their perception of body sizes for their spouse and children; 24% (13 of 54) of the children were perceived as being overweight.

**Table 1:** Descriptive characteristics of the respondents (n = 48)

	n (%)
Respondent shopping for self and child(ren)	44 (91.7)
Respondent shopping for self and spouse	25 (52.1)
Self-report of diagnosis by a physician for any household member:	
High blood pressure	7 (14.6)
Diabetes	1 (2.1)
Coronary heart disease	1 (2.1)
High cholesterol	4 (8.3)
Current smoker in household	7 (14.6)
Fair or poor health (respondent)	5 (10.4)
No physical activity (respondent)	28 (58.3)

Nearly every respondent reported eating fast-food at least once per month and 27% (13 of 48) ate fast-food once or twice per day (30 to 60 times per month; Table 2). A few persons hunted (n = 3) or farmed year-round (n = 4), and 17 persons gardened seasonally. The frequency of fast-food consumption was higher among the respondents who perceived themselves as being overweight than among the respondents who did not (Table 3).

**Table 2:** Frequency of dining at fast food restaurants (n = 48)

	Frequency
1 time per month	1
2 times per month	2
3 times per month	4
4 times per month	11
9 times per month	11
13 times per month	3
17 times per month	2
26 times per month	1
30 times per month	10
60 times per month	3

**Table 3:** Frequency of fast food consumption (per month) and perceived body size

	n	Mean frequency	P value
Respondent perceived as being overweight	27	18.8	
Respondent not perceived as being overweight	21	9.3	0.02
			
Anyone perceived as being overweight	37	15.5	
No one perceived as being overweight	7	13.1	0.16
			
Spouse perceived as being overweight	16	17.9	
Spouse not perceived as being overweight	9	12.3	0.36

Note: Analysis for anyone versus no one perceived as being overweight includes the sub-sample of respondents that have children in the household. Analysis for spouses includes the sub-sample of respondents that have a spouse in the household (including those with children).

**Figure 2:**
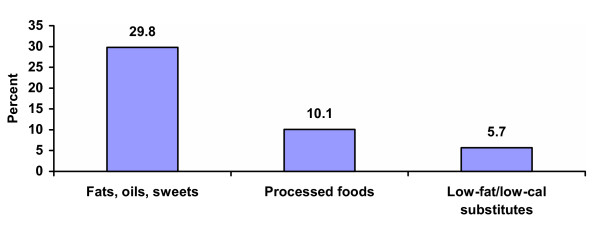
Proportion of dollars spent on 3 categories of food choices. Legend. Fats, oils, and sweets included food made of almost entirely fat or sugar such as regular soda, cookies, cakes, regular potato chips, and regular mayonnaise. Processed foods included food that was prepared with hydrogenated fats or cured meat such as bacon, hot dogs, bologna, and regular frozen meals. Low-fat/low-calorie substitutions included health-conscious choices, such as skim milk, diet soda, and baked chips, which indicated that the individual was attempting to lower his/her fat or caloric intake.

Overall, 30% of food purchases were allocated to fats, oils, sweets, 10% to processed foods, and almost 6% to low-fat/low-calorie substitutions (Figure 2). According to the silhouette data, households where no one was perceived to be overweight spent a smaller proportion of their food budget on fats, oils, and sweets than did households where at least one overweight person was perceived as overweight (21.2% vs. 31.7%, P = 0.10; Table 4). Households where the spouse was not perceived to be overweight spent proportionately less on fats, oils, and sweets (20.5% vs. 33.7%, P = 0.02) and more on low-fat/low-calorie substitutions (6.6% vs. 1.5%, P = 0.09) than did households where the spouse was perceived to be overweight. Finally, respondents who perceived themselves as being overweight spent a greater proportion of their food budget on processed food than did respondents who did not perceive themselves as being overweight (12.0% vs. 7.7%, P = 0.06).

**Table 4:** Percent of grocery receipt allocated to 3 categories of food choices by perceived overweight status of respondent and his or her household members.

	n	Fats, oils, and sweets Mean (SD)	Processed foods Mean (SD)	Low-fat/low-calorie substitutions Mean (SD)
Respondent not perceived as being overweight	21	26.5 (18.2)	7.7 (10.6)	6.9 (10.4)
Respondent perceived as being overweight	27	32.4 (16.3)	12.0 (15.1)*	4.7 (7.4)
				
No one perceived as being overweight	7	21.2 (17.7)	5.2 (12.4)	7.6 (14.4)
Anyone perceived as being overweight	37	31.7 (16.0)*	11.7 (13.8)	5.3 (7.9)
				
Spouse not perceived as being overweight	9	20.5 (16.5)	6.8 (12.5)	6.6 (9.3)
Spouse perceived as being overweight	16	33.7 (9.3)*	12.8 (16.7)	1.5 (3.2)*

*Wilcoxon two-sample exact test, one-sided P ≤ 0.10.

## Discussion

This simple method, although global in nature, is feasible and appears to have concurrent validity as a tool for dietary assessment as defined by associations with perceived body size by the shopper or the shopper's family. Although not all differences were statistically significant, they were in the hypothesized direction. For example, when the respondent did not perceive anyone in the household as being overweight, a higher proportion of the grocery bill was allocated to low-fat/low-calorie substitutions and a smaller proportion to processed foods (and to fats, oils and sweets which was statistically significant) than in households where any one was perceived as being overweight. When the respondents did not perceive themselves as being overweight, the allocation was similar to that in households where no one was perceived as being overweight; if the spouse was perceived as being overweight, allocations were also in the hypothesized direction. In the United Kingdom, a similar study found that households including mainly overweight individuals purchased food higher in fat than did households including mainly lean individuals (38% vs. 35% of total energy from fat, P = 0.001).[22]

Our findings are further substantiated by longitudinal analyses of dietary measures. Newby and colleagues concluded from 7-day dietary records that diets high in fruits and vegetables, low-fat dairy products, and whole grains and low in red and processed meats, fast-food, and soda are associated with smaller gains in BMI and waist circumference over a 25- to 26-month period.[23] In another study, "regular breakfast eaters" were less likely to develop diabetes or obesity than were those who did not eat breakfast regularly (Pereira MA, Van Horn L, Slattery M, Jacobs DR Jr, Ludwig DS, Reported Breakfast Habits and Incidence of Obesity and the Insulin Resistance Syndrome in Young Black and White Adults: The CARDIA Study American Heart Association's annual conference, March 6, 2003 Miami FL [Unpublished data]).

Comparable with other studies, our results showed that the respondents' frequency of fast-food consumption was related to overweight as determined from the silhouette drawings. Using 24-hour recall data from the Continuing Survey of Food Intakes by Individuals (CSFII 1994–1996), Paeratakul and colleagues found that adults and children who reported eating fast-food had higher intakes of energy, fat, saturated fat, sodium, and carbonated soft drinks, and lower intakes of vitamin A and C, milk, fruits and vegetables than did those who did not report eating fast-food (p < 0.001).[12] A second study of children aged 4–19 years from the CSFII 1994–1996 found that those who ate fast-food consumed more total energy, more energy per gram of food, more fat, more added sugar, and more sugar-sweetened beverages, and less milk, less fiber, and fewer fruits and nonstarchy vegetables than did those who did not report fast-food consumption.[24] A third study of adults ages 20 year and over from the CSFII 1994–1996 showed a significant relation between fast-food consumption and overweight status: adults who reported eating fast-food on at least 1 of 2 survey days had higher BMIs than did those who did not report eating fast-food on either survey day.[25]

The methods used in this study (i.e., collecting grocery receipts and asking about fast-food consumption) are feasible and have utility. Other researchers have also looked for alternatives to the common self-assessment dietary assessment tools (eg, 24-hour recall, food frequency questionnaire). For example, simple self-assessment tools based on food groups, designed for dietary assessment by nurses, have shown acceptable agreement with weighed food records.[26] These simpler methods, if proven valid and reliable, are more cost-effective than the standard tools, and are less burdensome for the respondent. Furthermore, the grocery receipt analysis is not limited by self-report bias or social desirability bias as are more detailed forms, such as the 24-hour recall, as some may be reluctant to report consumption of unhealthy food items.[6]

The limitations of this study must be noted. The data were derived from a small sample of volunteers and thus may not be generalizable to the population at-large, although the community where the data were collected is quite homogeneous. The categorization of the food purchases does not account for all foods, and does not distinguish between types of fat (e.g., saturated versus unsaturated). The study used a cross-sectional design, and the temporal relation between fast-food consumption and allocation of food purchases and perceived weight status cannot be determined. Given the brevity and anonymous nature of the questionnaire, we did not collect demographic or detailed information on other potential confounders of the associations (eg, education, TV-viewing habits). Furthermore, the adult silhouettes used to assess weight status do not have established validity and reproducibility for parental reports of children's body sizes, and proportion of food purchases is not a precise measure of food quantity in terms of caloric contribution.

In conclusion, we found that perceived overweight status of household members was related to the purchase of fats, oils, and sweets in the grocery store, and that perceived overweight status of the shopper was associated with the purchase of processed foods and frequency of consumption of fast food. Given the limitations of this study, we'd like to suggest further work to establish the validity and reproducibility of our methodology and our encouraging findings regarding its use.

## Competing interests

The author(s) declare that they have no competing interests.

## Note

Perceived overweight status = figure 5 or higher on silhouette drawings (see Appendix). Fats, oils, and sweets include food made of almost entirely fat or sugar, such as lard and regular soda. Processed foods include food that was prepared with hydrogenated fats or cured meat, such as bacon, hotdogs, bologna, and regular frozen meals; and low-fat/low-calories substitutions include health-conscious choices such as skim milk, diet soda, and baked potato chips, which indicated that the individual was attempting to lower his or her fat or caloric intake.

Analysis for anyone versus no one perceived as being overweight includes the sub-sample of respondents that have children in the household. Analysis for spouses includes the sub-sample of respondents that have a spouse in the household (including those with children).

## Appendix

Grocery Store Questionnaire Guide

After reading debriefing statement, secure their grocery receipt before asking these questions.

1. How many people would you say you were shopping for today?

a. How many kids? (ages please)

i. If kids, do they get the school lunch [yes / no], school breakfast [yes /no]

b. Any other adults? (ages please)

2. How often do you shop for groceries? (circle best response)

Once per month Two or three times per month

Once per week Twice per week Three or more times per week

3. How far do you live from this grocery store?

Within one mile within 5 miles within 10 miles

Within 20 miles within 40 miles within 60 miles more than 60

4. What made you chose this store today (rank in order if more than one reason)

prices location selection other

5. Do you think this shopping list is representative of what you often eat? yes / no

a. If no, please explain

6. How often do you eat fast food?

per month OR per week OR per day

7. Where else do you get your food? *What foods and How often for each:*

Farming:

Gardening:

Hunting:

Relatives Houses:

Restaurant (other than fast food):

8. How do you rate your health in general:

Poor Fair Good Very good Excellent

10. Has a doctor or nurse ever said that you or anyone in your house has

If yes, who (by relation only – no names)

a. High blood pressure yes / no

b. Diabetes yes / no

c. Heart disease yes / no

d. High cholesterol yes / no

If yes, who (by relation only – no names)

11. Does anyone in your house smoke? yes / no

12. Do you walk or do some other form of activity for exercise? yes / no

If Yes,

a. How many days per week usually?

b. How many minutes usually?

And finally, where do you think you fall in terms of body shape compared to these pictures?

Thanks, let's also put initials and ages by each other member of your household.
